# Estimation of Performance Characteristics of Analytical Methods for *Mycobacterium avium* subsp. *paratuberculosis* Detection in Dairy Products

**DOI:** 10.3389/fmicb.2019.00509

**Published:** 2019-03-15

**Authors:** Sophie Butot, Matteo Ricchi, Iker A. Sevilla, Lise Michot, Elena Molina, Maitane Tello, Simone Russo, Norma Arrigoni, Joseba M. Garrido, David Tomas

**Affiliations:** ^1^Nestlé Institute of Food Safety & Analytical Sciences, Nestlé Research Center, Lausanne, Switzerland; ^2^Istituto Zooprofilattico Sperimentale della Lombardia e dell’Emilia Romagna, National Reference Centre for Paratuberculosis, Brescia, Italy; ^3^Animal Health Department, NEIKER-Instituto Vasco de Investigación y Desarrollo Agrario, Bizkaia Science and Technology Park, Derio, Spain

**Keywords:** MAP, milk, sensitivity, trueness, LOD, culture, PCR, phage

## Abstract

Paratuberculosis is a chronic enteric infection, caused by *Mycobacterium avium* subsp. *paratuberculosis* (MAP), affecting virtually all ruminants as well as other animals. MAP is also suspected to be involved in the etiology of some human diseases, like Crohn’s disease and others. In surveillance studies, different analytical methodologies were employed to detect MAP, showing different results and incidence in dairy products. The aim of this study was to evaluate the performance characteristics of three analytical methods [culture, quantitative PCR (qPCR) and peptide-mediated magnetic separation (PMS) phage-based assay] for MAP detection in raw, heat-treated and powdered milk. The methods were evaluated according to performance characteristics defined for qualitative methods in ISO 16140-2:2016. To estimate sensitivity (including trueness) and LOD, 720, and 900 test portions, respectively, were blind tested by two laboratories. Considering all matrices, different sensitivities, expressed as the percentage of positives from the total of true positive test portions, were obtained for IS900 qPCR (94%), f57 qPCR (76%), culture (83%), and PMS-phage (40%). Trueness, expressed as results correctly assigned (including positive and negative) to the reference value, was 93% for the IS900 qPCR method, 89% for culture and 49% for the PMS-phage. The LODs obtained in this study were similar to the LODs previously published for cultural and qPCR methods. However, for the PMS-phage method, the obtained results showed higher LOD values compared to the limited data available in the scientific literature. Our results highlight that while the PMS-phage assay is workable in pure liquid culture for estimation of MAP counts, its usage for surveillance of dairy matrices should be treated with a lot of caution as performance characteristics obtained were lower than for the two other methods tested. qPCR and culture are the most appropriate methods to detect MAP in milk-based matrices according to ISO 16140 methodology. Cultural techniques are considered the gold standard for detection of viable MAP, but qPCR, which is widely used in analytical and surveillance studies, can be considered a suitable and recommendable alternative to cultural methods for screening, if confirmation of MAP’s viability is not requested.

## Introduction

Paratuberculosis or Johne’s Disease is a chronic enteric infection affecting virtually all ruminants as well as other animals (Sweeney et al., 1992). The disease is caused by *Mycobacterium avium* subsp. *paratuberculosis* (MAP), a member of the *M. avium* complex group. MAP is also suspected to be involved in the etiology of some human diseases, like Crohn’s disease and others. More precisely, a zoonotic role of MAP has not been definitively proven, but, on the other hand, it has never been ruled out (Chiodini et al., 2012; More et al., 2017). The described potential route of human exposure to MAP is the consumption of contaminated dairy products such as raw milk and cheeses, waters, and contaminated beef (Eltholth et al., 2009; Gill et al., 2011; Lorencova et al., 2014; Berthold-Pluta et al., 2015; Savi et al., 2015; Waddell et al., 2016). Numerous studies reported the presence of MAP in raw and pasteurized cows’ milk, as well as in other dairy products at retail level such as milk powder (Weber et al., 2008; Eltholth et al., 2009; Okura et al., 2012; Botsaris et al., 2016; Acharya et al., 2017). In these surveillance studies, different analytical methodologies with performance characteristics not evaluated by standardized protocols for food microbiology have been employed. Consequently, thoroughly validating the techniques used to detect the presence of MAP in these products is pivotal.

Historically, the only methods available for the detection of MAP in milk and other dairy products were the cultural assays, both in liquid and solid media (World Organization for Animal Health, 2014). Different approaches were proposed to inactivate the competing microflora present in the matrices which can affect the cultivability of MAP (Dundee et al., 2001). However, the main drawback of cultural methods is the extremely slow growth rate of MAP for the primary isolation, which can take up to 4 months for bovine strains and even longer for the ovine isolates (World Organization for Animal Health, 2014), making these methods extremely time demanding.

For these reasons, in the last 15–20 years, molecular methods (e.g., PCR/qPCR) aimed at detecting MAP DNA in various food and clinical samples have been regularly used (Möbius et al., 2008). Among the MAP DNA targets available, the most popular is the insertion sequence 900 (IS900) (Möbius et al., 2008). Notably, PCRs targeting this genetic element are the most sensitive because this sequence is present in multiple copies in the genome of MAP (in a range between 16 and 20 copies, depending on the strain) (Slana et al., 2008). The next most popular PCR target is the f57 sequence, which has been, so far, found only in MAP and represents a unique target making it the best choice when a specific test to confirm MAP identity is required (Vansnick et al., 2004). However, f57 element is present in only one copy in the genome of MAP, reducing the sensitivity of PCRs targeting this sequence compared to those targeting IS900 (Möbius et al., 2008).

More recently phage-based assays, coupled or not with peptide-mediated magnetic separation (PMS) for MAP cell capture, have been developed for the detection of MAP in milk (Stanley et al., 2007; Foddai et al., 2009, 2010a, 2011; Botsaris et al., 2013; Gerrard et al., 2018). This method is based on the ability of mycobacteriophage D29 to infect mycobacteria and has been used to detect viable MAP in dairy products (Botsaris et al., 2013, 2016; Gerrard et al., 2018). To the best of the authors’ knowledge, none of the methods previously or currently applied for the surveillance of MAP in dairy matrices, have been submitted to a rigorous evaluation of performance characteristics using a reference standard. This study has been performed considering the technical procedure included in ISO 17468 (International Organization for Standardization, 2016c), in particular the methods evaluation study, by assessing the methods applicability in different milk matrices (in artificial contamination conditions) and estimating performance characteristics as stated in the ISO 16140-2 (International Organization for Standardization, 2016b), which represents the recommended approach for the validation of the alternative methods in food microbiology. In the absence of a reference method for MAP detection in dairy products, the study clauses have been adapted to compare methods against a reference value.

The aim of this study was to evaluate the performance characteristics of three methods for MAP detection in raw milk, heat-treated milk and milk powder. The methods assessed were: culture (Dundee et al., 2001), IS900 qPCR (Donaghy et al., 2011) and f57 PCR (Ricchi et al., 2014), and PMS phage-based assay (Foddai and Grant, 2017). The methods were evaluated according to the technical protocol for qualitative methods included in ISO 16140-2:2016 (International Organization for Standardization, 2016b). Specifically, the analytical sensitivity and the limit of detection were calculated using artificially spiked test portions generated in one laboratory and submitted to two independent laboratories for blind analysis.

## Materials and Methods

### Preparation of Test Portions

Four reference strains of MAP (ATCC 19851; ATCC 43015; DSM 44133; and DSM 44135), were used to spike the test portions. Each freeze-dried MAP strain was grown as described by Peterz et al. (2016) to produce the reference and working stocks.

Prior to enumeration and inoculation, MAP cultures were declumped by filtration according to Serraino et al. (2017). To evaluate the quantity needed to spike the milk test portions, MAP pure cultures, previously declumped, were enumerated by microscopic examination using a Neubauer-improved counting chamber (depth of 0.02 mm), by Phage amplification assay as described by Foddai and Grant (2017) and by qPCR as described below. Prior to qPCR, the DNA was extracted from 1 ml of declumped MAP culture using Adiapure^TM^ kit (Bio-X Diagnostics, Rochefort, Belgium) according to manufacturers’ instructions. Milk test portions were spiked with declumped MAP cells by performing different 10-fold dilutions according to the estimated level of inoculum required.

Three different milk matrices were used in the study: heat-treated milk (four sources), milk powder (five sources), and raw milk (four sources). For heat-treated milk, commercial whole pasteurized, UHT whole, semi-skimmed and skimmed milk samples were purchased from Swiss, French, and Spanish supermarkets. For skimmed milk powder, three items typically used as constituent of culture media for microbiological methods (Sigma, St. Louis, MO, United States; Merck, Kenilworth, United States; Oxoid, Basingstoke, United Kingdom) and two commercial items obtained from Swiss supermarkets were used. Skimmed milk powder samples were reconstituted in sterile water at a ratio 1:10 (w:v). Raw bovine milk was collected from four Friesian farms from the Basque Country (Northern Spain) participating in an experimental paratuberculosis control program since 2006 (Garrido et al., 2013). The program includes vaccination of the replacement, annual blood and fecal sampling and monitoring of slaughtered animals. These four farms have been MAP-negative for at least the last five years. Milk samples were also analyzed by qPCR and confirmed to be negative by the official control laboratory.

Test portions (50 ml each) were distributed in centrifuge tubes, spiked (when required), randomly coded, frozen at −20°C and delivered to the participating laboratories. All test portions were confirmed frozen at arrival and stored frozen until analysis. All laboratories analyzed the test portions in blind within 4 weeks of receipt using cultural, qPCR (IS900 and f57) and PMS-phage methods.

The concentration of MAP cells in raw milk, heat-treated milk and reconstituted milk powder test portions after spiking was estimated by using the Most Probable Number (MPN) enumeration technique described in ISO 7218:2007 (International Organization for Standardization, 2007). For each test portion, three tubes from at least three different 10-fold dilutions were inoculated with 100 μl each onto Herrold’s Egg Yolk medium containing 2 mg/l of Mycobactin J (HEYM) (Becton Dickinson, Franklin Lakes, NJ, United States) and incubated at 37°C ± 1°C for up to 18 weeks. Colonies were confirmed by f57 qPCR as described in the analytical methods section. However, for the LOD study in raw milk, MPN determined by IS900 qPCR, which showed a good correlation with cultural method in previous samples, was used to calculate the reference values instead of the MPN by culture.

Homogeneity and stability studies to assess MAP detectability among replicates and during storage time were performed for every batch of spiked test portions at levels in which positive results were expected. For homogeneity, at least 10 test portions from each batch were analyzed at time 0 by cultural and IS900 qPCR methods as described in the analytical methods section. Stability was performed on test portions during a storage period of 4 weeks at −20°C. Every week, at least three test portions were defrosted at 4°C and analyzed by the cultural method.

Three non-spiked test portions of each batch were analyzed by cultural and qPCR methods to check for the absence of MAP.

### Sensitivity Study

A total of 720 test portions were prepared, including 240 test portions (120 per laboratory) of each milk type (heat-treated milk, milk powder, and raw milk) and comprising four milk items per milk type described previously. For each food item, 40% of the test portions were not inoculated (L_0_), 20% inoculated at low level, between 2 and 4 Log_10_/50 ml (L_1_) and the remaining 40% inoculated at high level (L_2_), between 3 and 5 Log_10_/50 ml (Table 1).

**Table 1:** Sensitivity study experimental design.

Milk type	Milk item	Strain	Number of test portions per inoculation level						Analytical method
			L_0_		L_1_		L_2_		
			Lab A	Lab B	Lab A	Lab B	Lab A	Lab B	
Heat-treated milk	UHT whole milk Spain	DSM 44133	4	4	2	2	4	4	Culture
			4	4	2	2	4	4	qPCR
			4	4	2	2	4	4	PMS-phage
	UHT semi-skimmed milk France	DSM 44135	4	4	2	2	4	4	Culture
			4	4	2	2	4	4	qPCR
			4	4	2	2	4	4	PMS-phage
	UHT skimmed milk France	ATCC 43015	4	4	2	2	4	4	Culture
			4	4	2	2	4	4	qPCR
			4	4	2	2	4	4	PMS-phage
	Pasteurized whole milk Switzerland	ATCC 19851	4	4	2	2	4	4	Culture
			4	4	2	2	4	4	qPCR
			4	4	2	2	4	4	PMS-phage
Powder milk	Skimmed milk powder Oxoid	DSM 44133	4	4	2	2	4	4	Culture
			4	4	2	2	4	4	qPCR
			4	4	2	2	4	4	PMS-phage
	Skimmed milk powder Swiss supermarket 1	DSM 44135	4	4	2	2	4	4	Culture
			4	4	2	2	4	4	qPCR
			4	4	2	2	4	4	PMS-phage
	Skimmed milk powder Sigma	ATCC 43015	4	4	2	2	4	4	Culture
			4	4	2	2	4	4	qPCR
			4	4	2	2	4	4	PMS-phage
	Skimmed milk powder Swiss supermarket 2	ATCC 19851	4	4	2	2	4	4	Culture
			4	4	2	2	4	4	qPCR
			4	4	2	2	4	4	PMS-phage
Raw milk	Raw milk 1	DSM 44133	4	4	2	2	4	4	Culture
			4	4	2	2	4	4	qPCR
			4	4	2	2	4	4	PMS-phage
	Raw milk 2	DSM 44135	4	4	2	2	4	4	Culture
			4	4	2	2	4	4	qPCR
			4	4	2	2	4	4	PMS-phage
	Raw milk 3	ATCC 43015	4	4	2	2	4	4	Culture
			4	4	2	2	4	4	qPCR
			4	4	2	2	4	4	PMS-phage
	Raw milk 4	ATCC 19851	4	4	2	2	4	4	Culture
			4	4	2	2	4	4	qPCR
			4	4	2	2	4	4	PMS-phage

Results from each laboratory were compared with the reference values (positive for spiked test portions and negative for non-spiked test portions) according to the following table and equations to determine the performance characteristics (International Organization for Standardization, 2016b):

**Table:** 

	Reference value positive	Reference value negative
Method positive (detected)	+/+ Positive Agreement (PA)	−/+ Positive Deviation (PD)
Method negative (not detected)	+/− Negative Deviation (ND)	−/− Negative Agreement (NA)

Sensitivity (SE), which was estimated as:

(1) $$\text{SE}=\frac{\left(\text{PA}+\text{PD}\right)}{\left(\text{PA}+\text{ND}+\text{PD}\right)}\times 100$$

Trueness (T), which was estimated as:

(2) $$\text{T}=\frac{\left(\text{PA}+\text{NA}\right)}{\left(\text{PA}+\text{ND}+\text{PD}+\text{NA}\right)}\times 100$$

Acceptability Limits (AL), which was estimated as the AL = ND-PD

Acceptability limits are the maximum acceptable difference between the reference value of a test portion and an individual result obtained when applying the analytical method (International Organization for Standardization, 2016a). The AL is not met when the observed value is higher than the maximum AL. Based on the AL and the additional information available (e.g., origin of the deviations) the alternative method is regarded as not fit for purpose for the category or categories involved.

For an unpaired study (results from each method are obtained from different test portions from the same sample), maximum limits are AL ≤3 for a single food type and AL ≤5 when three food types are tested (i.e., raw milk, heat-treated milk, and milk powder) (International Organization for Standardization, 2016b).

### LOD Study

A total of 900 test portions were prepared including 300 test portions (150 per laboratory) of each milk type (heat-treated milk, milk powder, and raw milk), with one item per milk type in this LOD study. For each food item, laboratory and method, five test portions were not spiked (L_0_), 20 test portions were spiked with a different MAP strain at low level (L_1_); 20 test portions at intermediate level (L_2_); and five test portions at high level (L_3_). Fractional results were expected at least for the L_2_ or L_3_ (Table 2).

**Table 2:** LOD study experimental design.

Milk type	Milk item	Strain	Number of test portions per inoculation level								Analytical method
			L_0_		L_1_		L_2_		L_3_		
			Lab A	Lab B	Lab A	Lab B	Lab A	Lab B	Lab A	Lab B	
Heat-treated milk	UHT whole milk	DSM 44135	5	5	20	20	20	20	5	5	Culture
			5	5	20	20	20	20	5	5	qPCR
			5	5	20	20	20	20	5	5	PMS-phage
Milk powder	Skimmed milk powder Merck	DSM 44133	5	5	20	20	20	20	5	5	Culture
			5	5	20	20	20	20	5	5	qPCR
			5	5	20	20	20	20	5	5	PMS-phage
Raw milk	Raw milk 1	ATCC 19851	5	5	20	20	20	20	5	5	Culture
			5	5	20	20	20	20	5	5	qPCR
			5	5	20	20	20	20	5	5	PMS-phage

Limit of detection 50% (LOD_50_) or 95% (LOD_95_) allows the estimation of MAP concentration, obtained with each method, for which the probability of detection is 50 or 95%, respectively.

Values for LOD_50_ and LOD_95_ were estimated using the complementary log-log model (Wilrich and Wilrich, 2009) considering the number of positive (detected) and negative (not detected) results for each method, food type and level of inoculation.

### Analytical Methods

#### Cultural Method

The cultural method applied was similar to previously used methods (Dundee et al., 2001; Grant et al., 2002; Donaghy et al., 2008; Taddei et al., 2008; Botsaris et al., 2010, 2013). Test portions (50 ml) were centrifuged 15 min at 2,500 *g* and the supernatants discarded. Raw milk pellets were resuspended in 25 ml of 0.75% Hexadecylpyridinium Chloride (HPC) (Sigma, St. Louis, MO, United States) and decontaminated at room temperature for 5 h. Heat-treated milk and reconstituted milk powder pellets were not decontaminated because significant background microflora was not expected. After decontamination, raw milk test portions were centrifuged again under the same conditions and the supernatants were discarded. The pellets obtained from heat-treated milk, milk powder, and decontaminated raw milk test portions were resuspended in 1 ml of PBS supplemented with 0.05% Tween 20 (Sigma, St. Louis, MO, United States).

The resuspended pellet was distributed in two slants of homemade HEYM medium supplemented with Chloramphenicol (30 mg/l) and Amphotericin B (50 mg/l) (HEYM/CAF) and in two slants of homemade or commercial (Becton Dickinson, Franklin Lakes, NJ, United States) HEYM supplemented with Amphotericin B (50 mg/l), Nalidixic acid (50 mg/l), Vancomycin (50 mg/l), and sodium pyruvate (4 g/l) (HEYM/ANV). The slants were incubated up to 18 weeks at 37 ± 1°C.

#### qPCR Method

Fifty ml of each milk test portion were centrifuged for 15 min at 2,500 *g*. Pellets were resuspended in 10 ml of sterile distilled water and MAP DNA was extracted using Adiapure^TM^ kit (Bio-X Diagnostics, Rochefort, Belgium) according to manufacturers’ instructions.

DNA was amplified using qPCR targeting IS900 and f57 sequences (Donaghy et al., 2011; Ricchi et al., 2014). Briefly, 5 μl of extracted DNA were transferred into the PCR reaction mixture (20 μl) containing 12.5 μl of Go Taq Probe qPCR Mix 2× (Promega Corporation, Madison, WI, United States), 300 mM of each primer (IS900 target or f57 target), 150 mM of the probe (IS900 target or f57 target), 2.5 μl TaqMan^®^ Exogenous Internal Positive Control mix (Life Technologies, Carlsbad, CA, United States) and 0.5 μl TaqMan^®^ Exogenous Internal Positive Control DNA (Life Technologies, Carlsbad, CA, United States). Amplification reactions were performed for each test portion using Applied Biosystems 7500, 7500 Fast Real-Time PCR, or StepOne Plus System (Life Technologies), with the following thermal cycling conditions: 1 cycle at 95°C for 10 min, 40 cycles at 95°C for 15 s, and 60°C for 1 min. For each qPCR run, positive and negative controls were also analyzed.

#### PMS-Phage Method

The procedure for coating magnetic beads with biotinylated-aMp3 (NYVIHDVPRHPA) or biotinylated-aMptD (GKNHHHQHHRPQ) peptides was based on Foddai and Grant (2017). Tosylactivated beads from two different vendors were used in each laboratory, 1 μm BcMag^TM^ Tosyl-activated magnetic beads (Bioclone Inc., San Diego, CA, United States) and 250 μl of MyOne^TM^ Tosylactivated Dynabeads^®^ (Life Technologies, Carlsbad, CA, United States) were coated with 0.25 mg/ml of each peptide following the instructions of Bioclone and with 1 mg of each peptide previously resuspended in 60 μl of molecular grade water according to Foddai and Grant (2017), respectively. Prior to PMS, 50 ml of milk were centrifuged as described previously for the qPCR and the cultural methods. The pellet was then resuspended in 1 ml of PBS containing 0.05% Tween 20 (Sigma, St. Louis, MO, United States). The PMS was carried out on one ml of concentrated test portion with 5 μl of biotinylated-aMp3 peptide- and 5 μl of biotinylated-aMptD peptide-coated magnetic beads using the environmental program of the Dynal BeadRetriever (Life Technologies, Carlsbad, CA, United States) or manually using the Dynal magnetic particle concentrator (MPC)-S as previously described (Foddai and Grant, 2017). For each PMS run, negative and positive controls were run alongside the milk samples.

Following the PMS, the test portions were subjected to the phage amplification assay according to Foddai and Grant (2017). Negative and positive controls were included in every phage amplification assay.

After the PMS-phage assay, plaques were counted and one to 10 plaques per plate were harvested immediately after the overnight incubation and pooled to extract the MAP DNA using Zymoclean^TM^ Gel DNA recovery Kit (Zymo Research, Irvine, CA, United States) according to manufacturer’s instructions, followed by qPCR targeting IS900 as described above.

## Results

### Sensitivity Study

**FIGURE 1:**
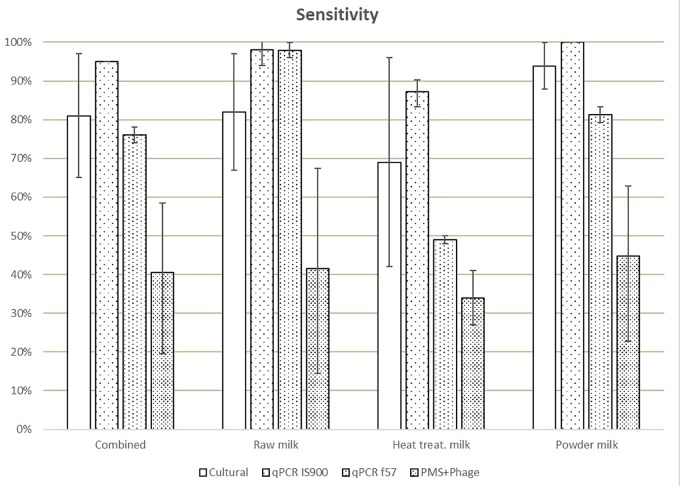
Means of the sensitivity values obtained in each laboratory for all dairy categories and methods. Error bars show the values obtained by laboratory A and laboratory B.

Different sensitivities, expressed as the percentage of positives from the total of true positive test portions (spiked samples), were obtained for IS900 qPCR (94%), f57 qPCR (76%), culture (83%), and PMS-phage (40%), when comparing all matrices (Figure 1). The lowest sensitivity results were obtained for heat-treated milk (35–87%) while the highest sensitivity for milk powder (45–100%) taking into consideration all the analytical methods. These differences are mainly associated to the high number of ND detected in both laboratories mainly in heat-treated milk test portions; 75 ND were observed out of 144 test portions of spiked heat-treated milk with 29 ND out of 48 spiked test portions from L_1_ and 46 ND out of 96 spiked test portions from L_2_. No potential relationship (e.g., per laboratory, per food type) were found to explain this high number of ND.

**FIGURE 2:**
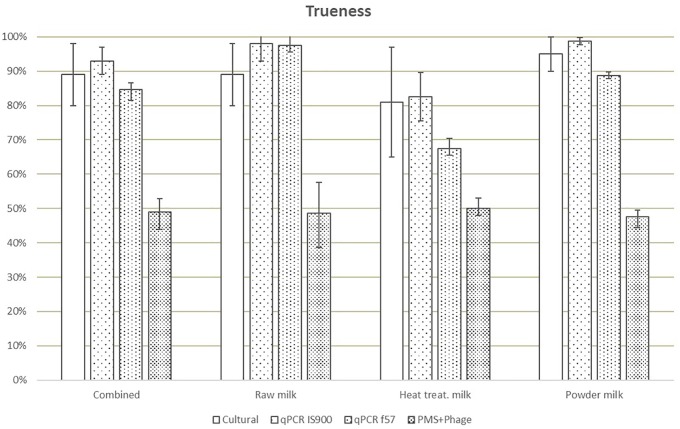
Means of trueness values obtained in each laboratory for all dairy categories and methods. Error bars show the values obtained by laboratory A and laboratory B.

Trueness, expressed as results (including positive and negative) correctly assigned considering the reference value, was highest for the IS900 qPCR method (93%) and lowest for the PMS-phage (49%) (Figure 2). The trueness value obtained for f57 qPCR was lower (85% versus 93%) than IS900 qPCR due to the lower number of PA.

Considering AL for all milk categories (Table 3), AL above the limits for cultural method are associated only to one laboratory due to the high number of ND. qPCR discordant results (4 versus AL ≤3) obtained by one laboratory were associated to false negative results on four heat-treated milk test portions spiked at low level (between <2.18 Log_10_ CFU/50 ml and 3.06 Log_10_ CFU/50 ml) with three different strains. For the PMS-phage method, results beyond the AL (i.e., >3) were observed in both laboratories and all three dairy categories and were associated to a high number of ND for all inoculum levels, food items and strains analyzed. Only laboratory A obtained acceptable results with raw milk but still with a high number of ND (10) and a relative high number of PD (7).

**Table 3:** Acceptance limits of the results obtained with all methods and dairy categories tested for the sensitivity study.

Milk category	Cultural		IS900 qPCR		f57 qPCR		PMS-phage		Max. AL
	Lab A	Lab B	Lab A	Lab B	Lab A	Lab B	Lab A	Lab B	
Raw milk	**1**	8	**0**	**0**	**0**	**0**	**3**	20	3
Heat-treated milk	**1**	14	4	−4	12	12	13	17	3
Milk powder	**0**	**3**	**0**	**−1**	4	5	4	18	3
Combined	**2**	25	**4**	**−5**	16	17	20	54	5

*Numbers in bold show the results within AL.*

*Mycobacterium avium* subsp. *paratuberculosis* was detected in all test portions tested for homogeneity. Only one out of 12 results from the raw milk test portions was not available due to overgrowth of background flora. The stability results were positive for all test portions of heat-treated milk and raw milk stored up to 4–6 weeks. For milk powder, one test portion (Skimmed milk powder spiked with strain reference DSM 44135) was positive after 3 weeks of storage but negative after 4 weeks. Despite that, this set of test portions showed positive results for the sensitivity study by both laboratories, which processed the test portions within three weeks after reception.

### LOD Study

Prior to test portion preparation for LOD study, the amount of MAP pure culture needed for spiking the test portions to reach the desired level for each milk type was determined by several rapid methods as shown in Table 4. Overall, the lowest enumeration values were obtained with the phage amplification assay followed by the Neubauer counting chamber, the f57 qPCR and the IS900 qPCR.

**Table 4:** *Mycobacterium avium* subsp. *paratuberculosis* inoculation levels L_0_, L_1_, L_2_, and L_3_, calculated from pure culture enumerations, in milk test portions prepared for the LOD study expressed in Log_10_ cells per 50 ml of milk.

Dairy product (Strain spiked)	Method used for enumeration in pure culture	Level of inoculation in milk calculated		
		L_1_	L_2_	L_3_
Raw milk	Neubauer counting chamber	3.26	3.96	5.66
(ATCC 19851)	IS900 qPCR	4.02	4.72	6.42
	f57 qPCR	3.52	4.22	5.92
	Phage amplification assay	2.27	2.97	4.67
Heat-treated	Neubauer counting chamber	3.48	3.78	5.48
milk	IS900 qPCR	3.91	4.21	5.91
(DSM 44135)	f57 qPCR	3.64	3.94	5.64
	Phage amplification assay	2.80	3.10	4.80
Milk powder	Neubauer counting chamber	4.13	4.83	5.83
(DSM 44133)	IS900 qPCR	4.80	5.50	6.50
	f57 qPCR	4.45	5.15	6.15
	Phage amplification assay	2.82	3.52	4.52

After spiking, the reference values were determined by MPN to calculate LOD (Table 5).

**Table 5:** Estimation of the MAP spiking levels L_0_, L_1_, L_2_, and L_3_ in milk test portions prepared for the LOD study by MPN technique expressed in log MPN/50 ml.

Dairy product (Strain spiked)	Method to be tested	L_0_	L_1_	L_2_	L_3_
Raw milk	Culture	0	3.88	4.88	5.18
(ATCC 19851)^1^	IS900 and f57 qPCR	0	3.18	3.88	5.18
	PMS-phage	0	3.18	3.88	5.18
Heat-treated	Culture	0	2.32	3.32	5.02
milk	IS900 and f57 qPCR	0	3.02	3.32	5.02
(DSM 44135)	PMS-phage	0	2.32	3.32	5.02
Milk powder	Culture	0	2.85	3.54	4.54
(DSM 44133)	IS900 and f57 qPCR	0	2.85	3.54	4.54
	PMS-phage	0	2.85	3.54	4.54

*Values are expressed considering the lowest dilution factor applied. ^1^Estimated by MPN qPCR.*

In general, the values are aligned but differences were observed between MPN and calculated values from pure culture enumerations because of the matrix effect and the analytical method used.

LOD_50_ and LOD_95_ were estimated considering combined results from both laboratories when possible (Table 6). In some cases, not enough fractional positives were obtained for an accurate estimation of the LOD. For the PMS-phage method, false positive results were obtained in non-spiked test portions from milk powder and raw milk, consequently these results have not been considered for the estimation of LOD.

**Table 6:** LOD_50_ and LOD_95_ results obtained for all methods and dairy categories tested and expressed in Log_10_ CFU/50 ml.

Method	Milk	LOD_50_	LOD_95_
Culture	Raw milk	4.20 (3.89; 4.51)^1^	4.83 (4.53; 5.15)^1^
	Heat-treated milk	1.80 (1.61; 1.98)^1,2^	2.44 (2.26; 2.61)^1,2^
	Milk powder	2.22 (1.93; 2.51)^1^	2.86 (2.57; 3.15)^1^
IS900 qPCR	Raw milk	2.74 (2.50; 2.98)	3.37 (3.13; 3.62)
	Heat-treated milk	2.98 (2.80; 3.17)	3.62 (3.44; 3.80)
	Milk powder	2.71 (2.47; 2.94)^1^	3.34 (3.11; 3.58)^1^
f57 qPCR	Raw milk	3.72 (3.49; 3.94)^1^	4.34 (4.11; 4.57)^1^
	Heat-treated milk	3.62 (3.32; 3.91)	4.26 (3.97; 4.54)
	Milk powder	3.15 (2.94; 3.34)	3.78 (3.58; 3.98)
PMS-phage	Raw milk	3.66 (3.44; 3.87)^1^	4.30 (4.08; 4.51)^1^
	Heat-treated milk	3.67 (3.35; 3.99)^1,2^	4.30 (3.99; 4.62)^1,2^
	Milk powder	3.16 (2.96; 3.36)	3.80 (3.59; 4.00)

*Between brackets, 95% Confidence Interval. ^1^Estimated values; ^2^combined results from both laboratories.*

The LOD results of the cultural method for raw milk were highly affected by the overgrowth of background microflora and by the estimation of the reference value by MPN using IS900 qPCR instead of the cultural method. For raw milk, the lowest LODs were obtained by IS900 qPCR, while for the other dairy products the lowest LODs were obtained by the cultural method.

## Discussion

Few studies, if any, use standard protocols to determine performance characteristics of methods which have been applied for the detection of MAP in dairy matrices. Consequently, the robustness of individual methods is difficult to establish and furthermore, comparison of MAP recoveries/detection between studies can be problematic. In this study, a recognized standard approach in food microbiology (International Organization for Standardization, 2016b) was used to estimate sensitivity, trueness and LOD of cultural, qPCR and PMS-phage methods in a range of dairy products. According to ISO 16140-1:2016 (International Organization for Standardization, 2016a), the sensitivity defines the ability of a method to detect the analyte; the trueness shows the closeness of agreement between the average of measured results of an infinite number of replicates and a reference value and the LOD estimates the concentration of the analyte giving a proportion (50 or 95%) of positive results for each method. To the best of our knowledge, this is the first study in the field of MAP reporting sensitivity and trueness data obtained following ISO 16140-2:2016 protocol (International Organization for Standardization, 2016b).

Overall, the IS900 qPCR was the most sensitive method. The sensitivity of the qPCR targeting f57 element was lower than that targeting IS900, which may be easily explained by the multiple copies of IS900 in the MAP’s genome, whereas only one copy of f57 element is present in this genome (Vansnick et al., 2004; Tasara and Stephan, 2005). The lowest variability between the two participating laboratories in terms of sensitivity was obtained for the qPCR methods. We believe this can be due to the use of standardized reagents (e.g., commercial kits for the DNA extraction and the qPCR reaction) in both laboratories. In contrast, the higher inter-laboratory variability obtained for the cultural method, may be explained by the use of different HEYM agar media: one laboratory used homemade HEYM media and the other laboratory used both homemade and a commercial media. According to the validation protocols followed, the PMS-phage assay showed the lowest sensitivity (<50%) across all dairy matrices and the highest inter-laboratory variability of all methods assessed.

As observed for the sensitivity, the trueness of the qPCR and the cultural methods consistently outperformed the PMS-phage method in both participating laboratories. Sensitivity and trueness results of qPCR and cultural methods were lower for heat-treated milk compared to the two other dairy products. One hypothesis to explain a lower performance of qPCR with this type of milk is that fat micro-droplets formed during milk heat treatment and homogenization could interfere with MAP-bead binding during bacterial concentration step of Adiapure kit. For the cultural method, this low performance was associated to a high proportion of ND in one laboratory. Regarding f57 qPCR, both laboratories reported similar results with a high percentage of ND for heat-treated milk not associated with low inoculation levels.

The LOD of cultural method estimated in this study for heat-treated and milk powder is similar to that reported by Akineden et al. (2014) for UHT milk. However, previous studies (Ruzante et al., 2006; Akineden et al., 2014) have reported LOD results for raw milk of 10 and 89 CFU/ml (corresponding to a range of 2.7 to 3.6 Log_10_ CFU/50 ml) showing better sensitivities than those obtained in this study. In this regards, it should be pointed out how our results were probably highly influenced by the presence of background microflora, which was already known for its potential to inhibit the growth of MAP (Taddei et al., 2008).

For dairy products tested in this study, LOD_50_ results obtained by qPCR are aligned with LOD reported in previous studies, ranging from 5 to 100 CFU/ml (corresponding to 2.4–3.7 Log_10_ CFU/50 ml) for IS900 (Rodríguez-Lázaro et al., 2005; Herthnek et al., 2008; Slana et al., 2008; Ricchi et al., 2016) and ranging from 10 to 100 CFU/ml (corresponding to 2.7–3.7 Log_10_ CFU/50 ml) for f57 (Tasara and Stephan, 2005; Slana et al., 2008). It should be noted that when the LOD by PCR or qPCR is compared to a reference value determined by cultural methods (such as MPN employed in this study), an overestimation of PCR sensitivity may be reported due to positive PCR reactions of non-viable microorganisms (Kralik et al., 2012).

For the PMS phage-based method, although some correlation has been established between CFU and PFU in MAP cells (Foddai et al., 2010b), full performance characteristics calculated following a standardized protocol are not available, making it difficult to compare the results (e.g., against other detection methods results). Results obtained in this study showed higher LOD values than the limited data available in the scientific literature. Foddai et al. (2010a) reported LOD ranging from 9 to 14.4 PFU/50 ml (corresponding to 0.95–1.16 Log_10_ PFU/50 ml) for a PMS phage-based method in raw milk but it is important to highlight that these results were obtained with reference values estimated in PFU rather than using a common standard reference (e.g., CFU or MPN) (Foddai et al., 2009); however, the experimental design used in this study did enable comparison of the PMS-phage method with a reference value.

Variants of the PMS phage-based method have been reported and applied by a limited number of groups (Foddai et al., 2009, 2010a,b, 2011; Botsaris et al., 2010, 2013, 2016; Foddai and Grant, 2017; Gerrard et al., 2018). In order to avoid technical competency issues with this method, a training was conducted in advance for the participating laboratories, by recognized users of the PMS-phage assay. Despite this, the PMS-phage consistently showed poorer sensitivity and trueness compared to the cultural and qPCR methods. Furthermore, calculated LOD_50_’s for heat-treated milk and milk powder were higher for the PMS-phage method compared to all other qPCR and cultural methods. From 126 non-spiked milk test portions, analyzed using the PMS-phage method by the two laboratories, 80 test portions generated phage plaques and 29 test portions were confirmed positive by qPCR. Some of these non-spiked milk test portions showed a high number of plaques (>300), which were not confirmed by qPCR, questioning the efficacy of the FAS treatment to inactivate exogenous phages.

In contrast, no PD for culture and f57 qPCR and only a low number of them for IS900 qPCR (eight PD detected in one laboratory) were observed among all these 126 non-spiked test portions.

While the PMS-phage assay is workable in pure liquid culture for estimation of MAP counts, its usage for surveillance of dairy matrices (raw and processed) should be treated with a lot of caution as performance characteristics obtained were inferior than for the two other methods tested.

Our study highlights that qPCR and culture are the most appropriate methods to detect MAP in milk-based matrices according to ISO 16140 methodology. Cultural techniques are considered the gold standard for detection of viable (and cultivable) MAP (Slana et al., 2008; Whittington et al., 2010). Despite the long incubation times required to detect the bacteria, cultural techniques are simple, robust, easy to implement and can guarantee the presence of viable MAP cells in the test portion by isolation and confirmation, including further identification steps when needed. Limitations may arise when dealing with difficult to grow or non-cultivable dormant strains (Whittington et al., 2004, 2011) as well as when contaminating microflora growth hampers the growth of MAP. On the other hand, PCR or qPCR, widely used in analytical and surveillance studies, can be considered a suitable and recommendable alternative to cultural methods for screening, if confirmation of MAP’s viability is not requested.

## Data Availability

All datasets generated for this study are included in the manuscript and/or the supplementary files.

## Author Contributions

DT and SB contributed to the design of the study, prepared the samples, and wrote the manuscript. MR and IS contributed to the design of the study, tested the samples, and wrote the manuscript. LM prepared the samples and helped to write the manuscript. SR, EM, and MT tested the samples and helped to write the manuscript. NA and JG supervised the study and helped to write the manuscript.

## Conflict of Interest Statement

The authors declare that the research was conducted in the absence of any commercial or financial relationships that could be construed as a potential conflict of interest.
